# The Prevalence of Pain in Patients Attending Sarcoma Outpatient Clinics

**DOI:** 10.1155/2011/813483

**Published:** 2011-05-05

**Authors:** P. Y. Kuo, J. T. C. Yen, G. M. Parker, S. Chapman, S. Kandikattu, I. Sohanpal, Y. Barbachano, J. E. Williams

**Affiliations:** The Royal Marsden, NHS Foundation Trust, London SW3 6JJ, UK

## Abstract

The prevalence of pain in patients with sarcoma is not well documented. We investigated this in outpatients at a tertiary cancer referral centre, assessing the adequacy of pain control and for risk factors leading to higher prevalence and severity of pain. 149 patients were surveyed. Patients with pain within the previous 7 days completed pain assessment tools (BPI, S-LANSS, PMI). 53% of patients had pain within the previous 7 days, and 25% had significant pain. Of those with pain, 63% was inadequately controlled and neuropathic pain was identified in 36%. Age, gender, tumour type, and the type of cancer treatment were not significant predictors of the prevalence or severity of the pain. Based on our results, patients with sarcoma should be actively screened for pain and have regular reviews of their analgesic requirements.

## 1. Introduction

The prevalence of pain in cancer patients was recently investigated in a meta-analysis involving 52 studies [1]. Studies from Europe, Asia, Australia, and USA have confirmed that cancer patients are repeatedly found to be in pain, both as in—and outpatients, with undertreatment of their pain—sometimes with no analgesia at all [2–8]. With a reported overall prevalence of 52–72% [1, 6, 9], irrespective of staging, this is an issue which needs to be addressed on a global scale. As pain prevalence is noted to vary amongst different cancer types [6], it is necessary to determine the prevalence of pain in specific cancer types, to raise awareness amongst clinicians, and to improve patient management.

Deandrea et al. [10] analysed 26 studies which used the Pain Management Index (PMI) [11], a recognised tool to evaluate the patient's analgesic strategy based on WHO guidelines [12]. They showed that 43% of patients had a negative PMI score—thus deemed undertreated. Cancer patients are living for longer and becoming long-term cancer “survivors.” Cancer diagnosis now also occurs earlier with the advancement of technology. Accurate assessment and treatment of cancer pain is quite rightly becoming more important, at an earlier stage, and for a longer period of time.

With regards to pain prevalence related specifically to sarcoma, there is very little documentation at all. Sarcoma is a relatively rare, heterogenous type of cancer of the connective tissue which may occur almost anywhere in the body. It is not gender or age specific. The true incidence of sarcoma is difficult to ascertain as tumours are generally documented with respect to their site of origin rather than tissue type. The Sarcoma Trust/Sarcoma UK quotes 3200 cases in the UK each year, or about 1% of all cancer diagnoses [13]. The Royal Marsden Hospital is a specialist referral centre for cancer in London where we have around 750 patients with sarcoma referred each year. 

We undertook a cross-sectional, prevalence study of pain in patients who attended the sarcoma outpatient clinics at the Royal Marsden Hospital. In patients who had any pain we attempted to specify the characteristics of their pain, including severity, duration of pain, and the presence of breakthrough pain. We questioned the adequacy of pain control by using the PMI, the perceived aetiology of their pain, and also recorded the incidence of neuropathic pain in these patients. Finally, we tested the hypothesis that particular risk factors were associated with significant pain. The study encompasses the first steps in the recommendations from the American Pain Society [14]—to screen for the presence of pain and complete comprehensive initial assessments—with the goal of improving the quality of cancer pain management.

## 2. Methods

Ethics approval was granted by the Ethics Committee of the Royal Marsden Hospita, and the study was registered with the Hospital Committee for Clinical Research prior to the study commencing. The research was carried out according to Good Clinical Practice guidelines [15] and recommendations in the Declaration of Helsinki [16].

Patients were recruited from all sarcoma outpatient clinics in our hospital over 6 months from May–November 2009.

Table 1 shows the inclusion criteria. The only exclusion criterion was if the patient's health would be compromised by participation in the study.

### 2.1. Recruitment

All eligible consecutive patients attending sarcoma outpatient clinics within the study period were contacted via post or telephone a week before their clinic appointment to inform them of the study. These patients were then approached by the research team (independent of the medical team) on the day of their appointment to consent to the study. Those recruited filled out a screening questionnaire, with the assistance of the research team.

### 2.2. Data Collection

The screening questionnaire collected the following information.

**Patient Demographics**

Age, treatment history (from the electronic patient records).

**Prevalence of Pain**

Pain in the previous 7 days, analgesic medication, patients who had pain then had further assessments of the characteristics and management of their pain.

**Assessment of Pain**

Duration of primary pain, presence of breakthrough pain, aetiology of pain (possible causes related to tumour pressure or infiltration, due to cancer treatment or noncancer pain), the Brief Pain Inventory (BPI) [17], researcher's evaluation as to whether this was nociceptive pain, neuropathic pain, or mixed nociceptive and neuropathic pain, and the self-assessment version of the Leeds Assessment of Neuropathic Symptoms and Signs pain scale (S-LANSS) [18]. The BPI contains a collection of Visual Analogue Scales (VAS) evaluating pain and its impact on daily function. S-LANSS assesses the neuropathic component by asking specific questions regarding the characteristics of the pain. Both are well-validated tools of pain self-assessment.

**Adequacy of Pain Management**

The Pain Management Index (PMI) [11] is another widely used tool to evaluate the congruence of the reported level of pain and the potency of the prescribed analgesic by comparison to the World Health Organisation analgesic ladder [12]. A negative score indicates inadequate analgesic for that level of pain.

**Risk Factors**

The following information was collected to investigate whether they were predictors of pain: age, gender, tumour type, staging, and treatment type (i.e., whether surgery, radio-, chemotherapy, or biological therapy had taken place). Binary logistic regression was used to identify risk factors for pain. The relationship between the severity of pain and the potential risk factors was investigated by using ordinal regression. The analyses were performed using SPSS version 17.

### 2.3. Outcomes

The primary outcomes were the prevalence of “significant pain” in all patients—pain score of 5.0 or more—and the proportion of patients with negative PMI, that is, pain that was inadequately managed. The pain score was a composite of the VAS for average pain, worst pain, least pain, and pain right now. The secondary outcomes were the prevalence of significant pain association with risk factors as mentioned above, the severity of pain associated with risk factors, and the proportion of patients with neuropathic pain as determined by using S-LANSS.

### 2.4. Poststudy Surveillance

Patients inadequately treated for pain, as determined by a negative PMI, were offered advice regarding ongoing pain management. They were given advice and contact details for our pain team, prescribed analgesia, advised to seek further medical input from their GP, or booked into our or patient's own pain management clinic as required.

## 3. Results

**Demographics**

149 subjects were recruited from 228 eligible patients. 79 did not participate due to reasons outlined in Table 2. Patients were targeted in 22 clinics from May to November 2009, 11 surgical, 7 medical oncology, 4 radiology. The age range of recruited patients spanned from 19 to 98 years, median 62 years. The gender ratio was 44% males (66/149) to 56% females (83/149). Tumour types are listed in Table 3. 33/149 (22%) of subjects had metastatic disease.

### 3.1. Pain Characteristics

**Prevalence**

The overall prevalence of any pain in the previous 7 days in the study population was 53% (79/149). Using the Visual Analogue Scale (VAS) to score their worst pain (range 1–10), 27 patients had mild pain (VAS 1–4), 28 patients had moderate pain (VAS 5–7), and 24 patients described severe pain according to the VAS (8–10). Significant pain (≥5.0 by the composite pain score) occurred in 25% of patients with pain (20/79), 95% confidence interval was 16–36.

**Pain Duration**

48/79 (61%) of patients who reported pain had background pain for over 3 months in duration. The majority of the remainder (32%) had pain for more than 1 week but less than 3 months. 3/79 (4%) had pain for less than 1 week. 3 patients described no background pain.

**Breakthrough Pain**

Breakthrough pain was problematic for 45 patients (57%). 31 patients (39%) had unresolved breakthrough pain for more than 3 months. 7 patients had breakthrough pain for more than 1 week, but less than 3 months. The remaining 7 patients had breakthrough pain for less than 1 week.

**Aetiology**

The aetiology of their pain was discussed with the patients. 7 patients felt that their pain was due to direct pressure from the tumour, 1 due to metastatic disease. 50 patients had pain due to anticancer treatment, and 21 had pain from noncancer causes—17 musculoskeletal, 2 abdominal, 2 unspecified.

**Adequacy of Pain Control**

50/79 patients (63%) who reported pain were found to have a negative PMI—that is, not adequately treated with regards to their pain.

**Neuropathic Pain**

From the description of their pain, 36 patients were assessed by the research team to have pain of nociceptive origin, 7 neuropathic, and 36 mixed nociceptive and neuropathic. Of those with a neuropathic component, 11 were taking adjuvant analgesics such as antidepressants or anticonvulsants—3 from the purely neuropathic group, 8 from the mixed nociceptive and neuropathic pain group.

By using the S-LANSS questionnaire, 29 patients (36%) fit the neuropathic pain criteria (i.e., score ≥12). 11 of these patients (38%) had significant pain with a composite pain score of ≥5 in the previous seven days. The worst VAS in the previous seven days for 14 patients (48%) was ≥8.

### 3.2. Risk Factors

Age (comparing those greater to less than 60 years old), gender, tumour type, and cancer treatment type were not implicated as predictors of pain by using the binary logistic regression analysis (Table 4). The severity of pain was also not found to be related to the above risk factors by analysis with ordinal regression/*χ* ^2^-testing (Table 5).

### 3.3. Poststudy Surveillance

Not all patients who had pain wanted poststudy surveillance. No intervention was performed in 35 patients (44%). 24/79 (31%) patients were given advice by the researchers and given contact details of our hospital pain team. 5 were advised to see their GP, 3 were prescribed an additional analgesic, and 13 were advised to liaise with their local pain services, including 3 in our own pain management clinics. Of the group for further contact with pain management services, 9 had breakthrough pain for more than 3 months and 9 scored positive for neuropathic pain using the S-LANSS questionnaire. 2 patients required referral back to their clinical team for further investigation of the source of their pain.

## 4. Discussion

In our paper, we found the prevalence of pain in our patients with sarcoma to be 53%, which confirms the overall figure quoted in the meta-analysis of 52 studies of pain prevalence in cancer patients [1]. With 25% of patients having significant primary pain, it was clear that the management of pain in these patients should be reassessed, and possibly revised. 22% of our patient group had metastatic disease, and this may be one reason for the ratio of our subjects with significant pain to be quite high, as patient with advanced disease were previously found to have a higher incidence of pain [1, 3, 4]. 

This high prevalence of pain was unfortunately accompanied by a similarly high incidence of inadequately treated pain, as seen by 63% of patients having a negative PMI. This again echoes current evidence that a high percentage of cancer patients are still being undertreated for pain [10], despite repeated guidelines from agencies such as the WHO [12, 19, 20] and the Expert Working Group of the European Association for Palliative Care [21], and assertions that 90% of cancer pain should be manageable [20, 22]. Of note, the PMI does not take into account adjuvant analgesics, so one should bear this in mind when considering the results.

We did not specifically question patients regarding pain syndromes, but rather any pain that they may have. However, it is concerning that 61% patients had pain for more than 3 months in duration. We know from other studies that undertreatment of pain may result in patients having increased morbidity [23, 24], increased prevalence of depression and anxiety [25, 26], decreased enjoyment of life [6, 27], poor sleep [28, 29], inability to self-care [6], poor concentration, and poor personal interactions [6, 30]. Evidence available supports the need for prompt treatment of patients with acute pain, to prevent neuroplasticity, chronic pain syndrome, and immune suppression [14]. It also has massive socioeconomic costs including loss in productivity [6, 31, 32], work days [25], and litigations [33].

Breakthrough pain was a difficulty for over half of our patients with pain. Again, this has ramifications regarding poor prognosis, reduced function, mood, and hospitalisations [34–37]. It should be identified and promptly treated with regular reviews as part of the care package.

Nearly two-thirds of patients with pain felt that it was as a result of anticancer treatment. With this in mind, clinicians ought to be more vigilant regarding repeated pain assessment and management alongside their anticancer treatments. Patients are known to be reluctant to draw attention to their pain due to many reasons [6, 23, 25, 38–40] and need to be educated to do so, without fear that it will compromise their anticancer treatment. One quarter of patients had pain from noncancer sources. This confirms figures from previous studies [41] and also from patients in other cancer groups [42–44], highlighting the importance of a holistic approach to patient care.

A third of the patients with pain were assessed by the research team to have a neuropathic component using S-LANSS. A high percentage of those who scored positive for neuropathic pain with the S-LANSS questionnaire rated their pain to be significant. Neuropathic pain clearly plays a considerable role in this patient group, and wider use of adjuvant analgesic therapy may be necessary to help to control the pain. 

We were unable to identify any predictors of presence of any pain or severity of pain in our patient group. This may be as a result of insufficient sample size, or be due to the aforementioned heterogeneity in sarcoma. There may also be other factors not analysed in the study. Due to the rarity of sarcoma, it would take some time to organise a larger study to confirm this. Suffice to say that it would be prudent for clinicians to be aware of pain as a potential problem in all patients with sarcoma.

## 5. Conclusion

We have shown pain to be a significant problem in patients who attend sarcoma outpatient clinics, irrespective of the stage of their disease. Highlighted issues include severity of pain, duration of pain, and presence of breakthrough pain. Treatment of pain is generally inadequate and requires a full initial assessment and regular reviews, alongside treatment of the cancer. This should be a holistic approach to ensure that patients who have pain from non-cancer causes are also evaluated and referred as appropriate. Frequently, a neuropathic component to the pain may make treatment strategies complicated and adjuvants or specialist input may be beneficial. Future studies into pain prevalence in patients with sarcoma would be worthwhile to identify risk factors. In the meantime, it is up to the clinician to be aware of the possibility of a patient in pain who may be wary of drawing attention to the problem, resulting in long-lasting consequences.

## Figures and Tables

**Table 1:** Inclusion criteria for study.

All patients attending sarcoma outpatients who were:	
(i) aged 18 and above,	
(ii) diagnosed with sarcoma,	
(iii) able to respond to an assessment written in English,	
(iv) able to provide informed consent to participate in the study.	

Also eligible were: patients who had received anticancer treatments (includes surgery, chemotherapy, radiotherapy and biological therapy) or were currently receiving anticancer therapy, and patients with advanced or metastatic disease.

**Table 2:** Reasons for excluding patients.

Reason for exclusion	Number of patients excluded
Missed at clinic	39
Refusal	16
Did not attend/cancelled	12
Histology benign	7
Histology unconfirmed sarcoma	3
Too upset to approach	1
Poor English	1

Total	79

**Table 3:** Types of tumour.

Type of tumour	Number of patients
Angiosarcoma	3
Chondrosarcoma	1
Ewings sarcoma	1
Fibrosarcoma	16
Liposarcoma	30
Osteosarcoma	3
Other	80
Rhabdomyosarcoma	2
Soft tissue sarcoma	13

Total	149

Other: Leiomyosarcoma (21), GIST (11), Pleomorphic (11), Spindle cell (7), Synovial (4), Dermatofibrosarcoma (4), Fibromyosarcoma (3), Alveolar soft tissue (1), GIST pancreas (1), Stromal (1), Myoxoid (1), Undifferentiated round cell (1), Peripheral nerve sheath (1), Endometrial stromal (1), Myxofibrosarcoma (1), Gastric (1), Small round cell (1), Mesenteric bowel (1), Epithelioid (1), Giant cell (1), and Other unspecified (6).

**Table 4:** Risk factors associated with presence of pain.

Risk Factor	*P* value
Age	.173
Gender	.322
Tumour type	All values > .05
Chemotherapy	.904
Biological therapy	.880
Radiotherapy	.445

Anticancer Surgery	.203
(i) Amputation	.792
(ii) Wide local excision	.091
(iii) Surgery for recurrence	.188

All *P* values >  .05; hence all are not significant.

**Table 5:** Risk factors assessed for prediction of pain severity.

Risk factor	*P* value
Age (60 years as cutoff)	.651
Gender	.964
Tumour type	.817
All surgery	.622
Amputation	.251
Wide local excision	.613
Surgery for recurrence	.680
Chemotherapy	.565
Biological therapy	.923
Radiotherapy	.959
Duration of pain	.198
Cause of pain	.743

All *P* values >  .05; hence all are nonsignificant.
